# Investigation of Resistive Switching in Cu/a-SiC/P^+^-Si Structure for Multilevel Nonvolatile Memory Applications

**DOI:** 10.3390/mi17030364

**Published:** 2026-03-17

**Authors:** Hehong Shao, Xiuwei Zhu, Xin Zhang, Wanting Zheng, Libing Zhang, Liangliang Chen

**Affiliations:** 1 School of Biomedical Engineering, National Engineering Research Center of Ophthalmology and Optometry, Eye Hospital, Wenzhou Medical University, Wenzhou 325027, China; shhh@wmu.edu.cn (H.S.); zhuxw@wmu.edu.cn (X.Z.); zhangxin@wmu.edu.cn (X.Z.); zwt@wmu.edu.cn (W.Z.); zhlb@wmu.edu.cn (L.Z.); 2 School of Electronic Science and Engineering, Nanjing University, Nanjing 210093, China

**Keywords:** RRAM, multilevel storage, multiple conductive filaments, compliance current, SiC

## Abstract

Here, the resistive switching characteristics in a Cu/a-SiC/P+-Si sandwiched structure are systematically investigated for multilevel nonvolatile memory applications. The formation of Cu conducting filaments is believed to be the switching mechanism through temperature-dependent testing. Four distinguished resistance states can be achieved in the Cu/a-SiC/P+-Si memory device through the modulation of suitable compliance current, which could be attributed to the formation of more conductive filaments when applying a higher compliance current during the Set process. In addition, these different resistance values can be easily distinguished and show reliable retention (~105 s), with the temperature even reaching 85 °C, which offers considerable potential for high-density RRAM applications.

## 1. Introduction

Progress in artificial intelligence and data-centric computing is creating a need for the exploration and pursuit of high-efficiency parallel computing methods and high-density storage equipment [1,2]. Resistive random-access memory (RRAM), one of the most promising forms of next-generation nonvolatile memory, has been extensively studied for in-memory and neuromorphic computing recently due to its small physical size, high speed, low power consumption, multistate programmability and compatibility with the standard complementary metal oxide semiconductor (CMOS) process [3,4,5,6,7]. To meet the demands of the Big Data era for massive data storage, researchers have employed various methods to increase the storage density of RRAM. Historically, scaling down of device dimensions as well as three-dimensional (3D) crossbar architectures has been employed to achieve greater storage density in RRAM. However, the complexity of the required fabrication processes hinders its practical implementation [8,9]. In comparison to the two methods, multilevel cell (MLC) technology provides a much simpler alternative for enhancing RRAM storage density. It achieves this by allowing multiple bits to be stored in a single cell, thereby eliminating the requirement to shrink the physical device. So, MLC functionality is a key advantage of RRAM, offering a promising pathway to significantly enhance memory storage density [10,11,12,13,14,15]. Numerous studies have reported methods for achieving multilevel storage capabilities in RRAM, typically by modulating the compliance current or reset voltage to attain controllable multilevel states in the devices [12,15,16]. This is because the resistance variability in RRAM primarily correlates with the dimensions or numbers of the formed conductive filaments (CFs) within devices, which can be obtained by adjusting the compliance current or the reset voltage [6,10].

It is noted that silicon carbide has special attractions for resistive switching memory due to the high thermal conductivity, wide band gap and compatibility with CMOS technology [17,18]. As a third-generation wide-band-gap semiconductor material, SiC-based RRAMs have the potential to achieve a larger memory window, thereby enabling access to a greater number of distinct resistance states. Zhong et al. have conducted extensive studies on SiC-based RRAMs with active metal electrodes. Their work indicated that the Cu/a-SiC/W [17] or Cu/a-SiC/Au [18] structured RRAM can exhibit an extremely high ON/OFF ratio, and they also conducted a preliminary exploration about their multilevel storage characteristic, while the mechanism by which the device achieved multilevel storage remains unclear.

In this paper, the multilevel storage capability of an amorphous silicon carbide (a-SiC) RRAM device with a Cu/a-SiC/P^+^-Si structure was deeply investigated by setting different compliance currents. Actually, good endurance as well as data retention characteristics of four distinguished and stable resistance states were achieved in the Cu/a-SiC/P^+^-Si device. Furthermore, we employed the refined dc voltage sweeping mode with 3 mV/s to explore the mechanism of the multilevel behavior, which can be explained by the simultaneous occurrence of both filament radial expansion and the generation of additional conductive filaments under higher compliance currents.

## 2. Experimental Details

**Figure 1:**
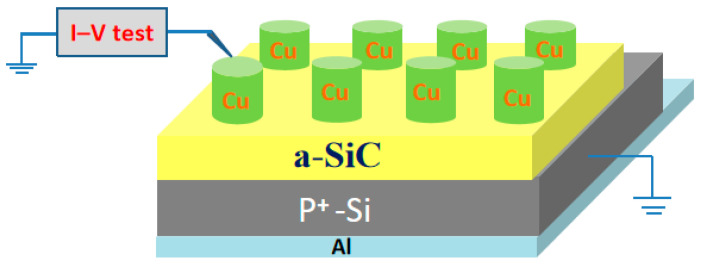
The structural diagrams of the Cu/a-SiC/P^+^-Si structure.

Figure 1 shows the structural diagrams of the Cu/a-SiC/P^+^-Si sandwiched-structure memory cells. Prior to deposition, the Si substrates were cleaned by a standard Radio Corporation of America (RCA) cleaning method. After that, the 30 nm thick a-SiC film was deposited on P^+^-Si substrate by the sputtering of the SiC target using a sputtering system with an RF power of 25 W at room temperature in an Ar atmosphere. Aluminum was also deposited at the back side of the Si substrate as the back electrode for better contact by electron beam evaporation. Then, 100 nm thick Cu top electrodes (TEs) were deposited on the surfaces of the film via RF magnetic sputtering through a shadow mask with diameters ranging from 100, 300, and 500 to 800 µm. The electrical measurements of the device were performed by using the Agilent B1500A semiconductor parameter analyzer (Agilent Technologies Inc., Santa Clara, CA, USA) with the Agilent 16440A pulse generator (Agilent Technologies Inc.). In all measurements, the bias voltage was applied to the Cu TE while the P^+^-Si BE was grounded. The temperature dependence of the I–V characteristics was examined in the Lake Shore CRX-4K system (Lake Shore Cryotronics, Inc., Westerville, OH, USA) under a vacuum of 5 × 10^−5^ Torr.

## 3. Results and Discussion

**Figure 2:**
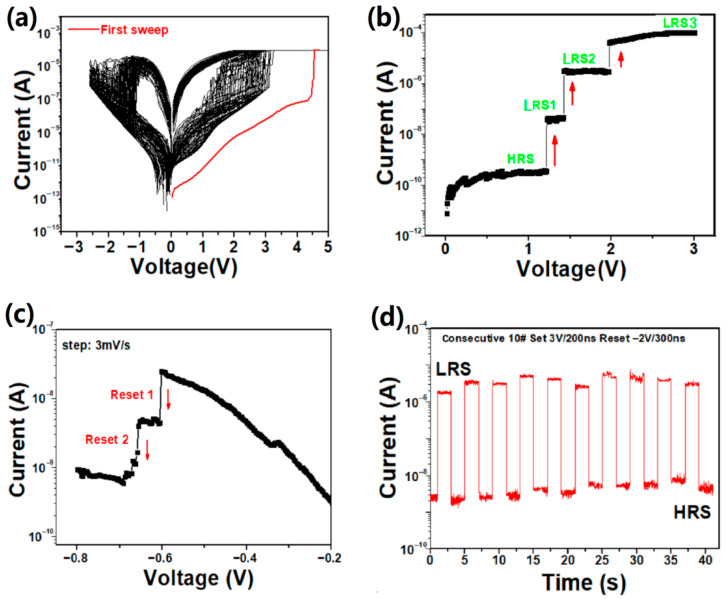
(**a**) Resistive switching I–V curves of the Cu/a-SiC/P^+^-Si device during the SET and RESET process after 100 cycles as marked by black lines and the first set is marked by red lines. (**b**) The refined current–voltage I–V characteristics of the Cu/a-SiC/P^+^-Si device under the dc voltage sweep mode with a very small increasing rate of 3 mv/step. It clearly shows the stepwise current switching in the on state. (**c**) The stepwise current switching in the off state with different reset voltages. (**d**) The real-time monitoring of the consecutive switching process with 10 cycles. The switching speed under a SET pulse of 3.2 V for 200 ns and a RESET pulse of −1.8 V for 300 ns, respectively.

The dc I–V curves of 100 consecutive cycles for the Cu/a-SiC/P^+^-Si device are depicted in Figure 2a. As shown in Figure 2a, the device presented a typical bipolar RS phenomenon. A forming process using relatively large bias (V_forming_ = 4.5 V) was required to initialize the devices. During this process, a compliance current (I_comp_) of 100 μA was used to prevent device cell destruction. When a positive voltage at about 2.4 V (the average value) was applied on the top electrode, the device switched from the high-resistance state (HRS) to low-resistance state (LRS) (denoted as SET process), and the current increase was larger than three orders of magnitude, which showed a large enough memory window to enable fast and reliable detection of the states of the memory cell [19]. After the Set process, the device remained at a low resistance value until the applied voltage reversed. The Cu/a-SiC/P^+^-Si could turn back to the HRS at about −1.8 V (denoted as RESET process). It was interesting to note that several multi-step switching behaviors were observed in the SET process, as seen in Figure 2a.

To obtain the refined stepwise curve, a dc voltage sweeping mode with a very small sweeping voltage incremental rate of 3 mV/step was performed. As observed in Figure 2b, the current increased abruptly at a voltage of about 1.3 V, which was called the Set I process. This indicated that the sample switched from the HRS to medium-resistance state (noted LRS1). As the voltage increased to about 1.6 V, the Set II process occurred when the device switched from LRS1 to a lower resistance called LRS2. The Set III process occurred until the voltage reached 2.1 V while the current reached I_comp_, and the sample switched from LRS2 to LRS3. According to Liu’s work, similar multi-step switching behaviors have also been reported in some other RRAM devices such as Cu/WO_3_/Pt [10], Al/CeO_x_/Pt [20], Cu/HfO_2_:Cu/n^+^-Si [21] and Ag/Ag-Ge-S/W [12], which might be related to the formation of multiple CFs in the Set process, which will be discussed later. Figure 2c shows that the device can also achieve different resistance states by adjusting the reset voltage. Typically, resistance states achieved through voltage modulation exhibit greater variability than those obtained via current regulation since they are highly dependent on the ruptured filament length which varies from cycle to cycle [6]. To achieve stable distinct resistance states, the multilevel storage characteristics of our device will be investigated by varying compliance currents subsequently. Figure 2d explores the switching speed of the Cu/a-SiC/P^+^-Si device. We carried out a pulse mode on the device. The switching speed was obtained under a SET pulse of 3.2 V for 200 ns and a RESET pulse of −1.8 V for 300 ns, determined by real-time monitoring for 10# consecutive Set/Reset switching processes, which indicated the fast switching speed of the Cu/a-SiC/P^+^-Si memory device.

**Figure 3:**
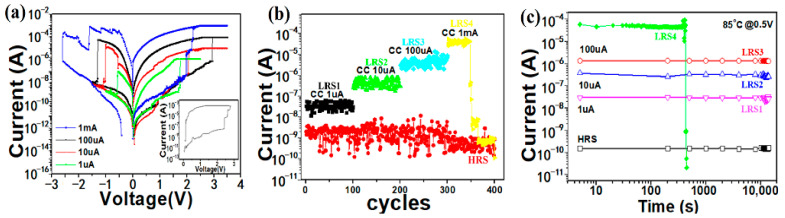
(**a**) The RS behavior in the Cu/a-SiC/P^+^-Si structure in different compliance currents. The inset shows that the device frequently fails to stay in the low-resistance state under 1 mA CC. (**b**) Endurance performance of the current on the HRS and LRSs. (**c**) Data retention of the LRSs in different compliance currents and the HRS @ 85 °C. The current values on the LRS and HRS were read at 0.5 V in each dc sweep.

Figure 3a presents the bipolar RS behaviors of the Cu/a-SiC/P^+^-Si memory device by setting different I_comp_ values. Through setting different I_comp_ values of 1 µA, 10 µA, 100 µA and 1 mA during the DC voltage sweep, the Cu/a-SiC/P^+^-Si device could be set to four different LRSs. It is worth pointing out that the Cu/a-SiC/P^+^-Si device still exhibited bipolar resistive switching characteristics when the compliance current was increased to 1 mA. However, the device was found to frequently fail to stay in the low-resistance state under a compliance current of 1 mA, as illustrated in the inset of Figure 3a. The distributions of the HRS resistance and four different LRS resistances (LRS1, 2, 3, 4) of the Cu/a-SiC/P^+^-Si device during 100# successive dc switching cycles are shown in Figure 3b, and the value of each resistance state was measured at a 0.5 V reading voltage. It can be found that the resistance states switched from HRS to LRS1, then from LRS1 to LRS2, and finally from LRS2 to LRS3, and each LRS resistance ratio was about 10 times. Although the device could successfully switch from LRS3 to LRS4 when the current compliance increased from 100 µA to 1 mA, it is evident that LRS4 automatically returned to the HRS after fewer than 50 cycles due to its inability to maintain the low-resistance state. Furthermore, determination of the data retention of the multilevel resistance states was performed at 85 °C and read at 0.5 V, as shown in Figure 3c. It can be seen that the HRS and the LRS1~LRS3 show good retention without obvious degradation while the device exhibits degraded retention performance at a compliance current of 1 mA. We can see that the LRS4 abruptly fails and reverts to the HRS at approximately 400 s. This phenomenon is likely attributable to two concurrent factors: the increasing number and the thickening of the conductive filaments (CFs) within the device as the compliance current rises, both of which contribute to a further reduction in resistance. According to Guo et al. [22], the filament temperature increases when a higher current passes through these low-resistance copper CFs, accelerating the migration of copper atoms within the filaments. The enhanced atomic migration promotes filament rupture, consequently causing the device to revert from the LRS to HRS spontaneously.

Hence, although the compliance current can be adjusted to control the number and the thickness of conductive filaments, leading to various low-resistance states, an excessively high compliance current will ultimately lead to device failure. For the device Cu/a-SiC/P^+^-Si, the three adjustable LRSs as well as the HRS resulted in four distinguishable switching levels, as demonstrated at compliance currents of 1 μA, 10 μA, and 100 μA, suggesting the potential application on multilevel memory.

**Figure 4:**
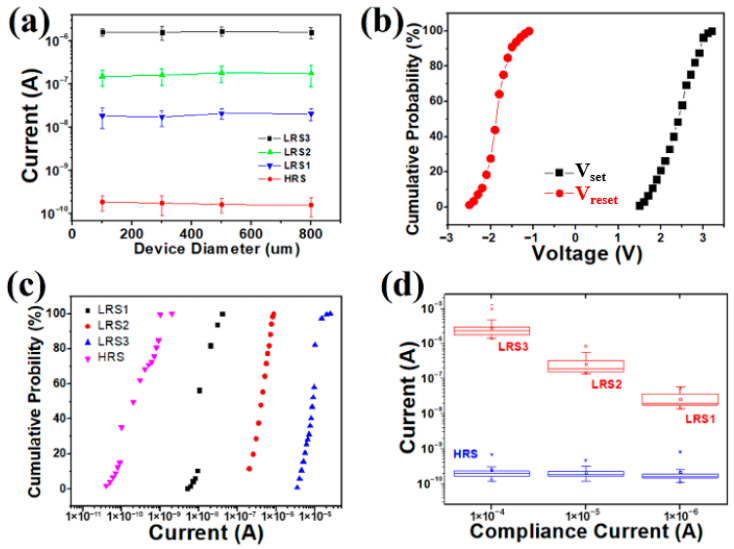
(**a**) The currents of the memory cell with different area scaling in the HRS and LRS. (**b**) Variation distribution of device-to-device V_set_ and V_Reset_ and (**c**) the current on LRS/HRS values. Data were obtained from 10 devices and each was run for 50 cycles. (**d**) Box plots of the current on different LRSs and HRSs read during DC cycles, using a 0.5 V DC read.

Several Cu/a-SiC/P^+^-Si devices were fabricated with the electrode diameter ranging from 100, 300, and 500 to 800 µm. We randomly selected ten devices including all of the four sizes to study the I–V behaviors as well as device-to-device uniformity (50 cycles for each device). As shown in Figure 4a, it is clear that both HRS and LRS currents exhibit small current variations as the electrode size increases from 100 to 800 µm, suggesting a local conductive filament. Figure 4b,c show the statistical distributions of the Set/Reset voltage and the currents on four resistance states across device to device, respectively. The Set/Reset voltage and the current on LRS/HRS values were read at 0.5 V in each dc sweep. As is seen, the V_set_/V_reset_ range of the Cu/a-SiC/P^+^-Si was 1.5~3.2 V/−1.2~−2.5 V, which indicates a relatively narrow distribution of both Set and Reset voltages. The current on LRS1 varies from 10^−9^ to 10^−8^ A; the current on LRS2 is confined to 10^−7^ A; and the majority of the current on LRS3 values are of the order of 10^−6^, with only a very few fluctuating around 10^−5^. The current on the HRS fluctuates between 10^−11^ and 10^−10^ A. Apparently, the four resistances show good stability with a satisfactory ON/OFF ratio (~10 times) between one another. The device-to-device coefficients of variation (standard deviations/mean value) for V_set_, V_Reset_, LRS1, LRS2, LRS3 and HRS are calculated to be 0.41, 0.28, 0.47, 0.38, 0.34, 0.43, respectively. The cumulative probability results show acceptable distribution, indicating the good device-to-device uniformity. Figure 4d is the box plot which shows cycle-to-cycle variation for the current on the four resistance levels (100 cycles for a memory). It can be observed that the Cu/a-SiC/P^+^-Si memory shows clear multilevel switching with distinct regions for each level, indicating the great potential for Cu/a-SiC/P^+^-Si memory to be used for multilevel switching.

As is known, local conductive filament formation and rupture modeling with the help of a redox reaction are commonly used to explain the switching behavior of RRAM [23]. For RRAMs with an oxidizable electrode (Cu or Ag), this kind of filament utilizes the composition of metal ions transferring from the oxidizable electrode to the inert electrode due to an electrochemical reaction [24,25]. Similarly, the formation and disruption of copper conductive filaments are related to the switching between the LRS and HRS in the Cu/a-SiC/P^+^-Si device. In addition, the multilevel storage behavior observed in the device can be explained by the formation of multiple conductive filaments within the device under a positive voltage bias [10,26].

**Figure 5:**
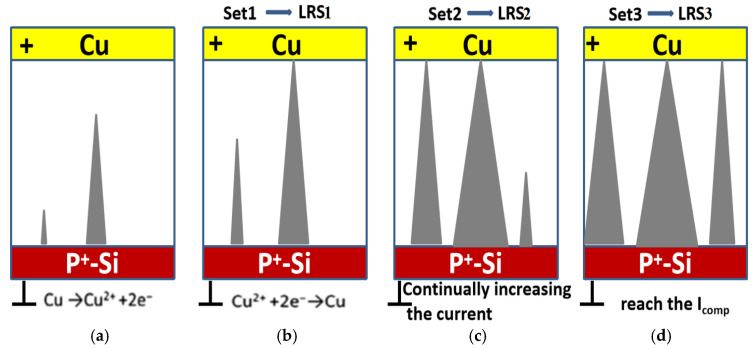
(**a**) Randomly distributed cone-shaped copper accumulations of varying sizes within the device. (**b**) The device switches from HRS to LRS1 due to the first CF connecting the top and bottom electrodes. (**c**) The device switches from LRS1 to LRS2 due to other CFs connecting the top and bottom electrodes. (**d**) The device transitions to its final LRS3 when the compliance current is reached.

Figure 5 illustrates the process of obtaining different resistance values by changing the compliance current in the presence of multiple conductive filaments within the device. When a positive voltage is applied to the copper electrode of the Cu/a-SiC/P^+^-Si device, the chemically active copper undergoes an electrochemical reaction under the electric field, generating copper ions. The reaction equation is as follows:

Cu − 2e^−^ → Cu^2+^

Driven by the forward electric field, Cu^2+^ ions migrate to the cathode and are reduced to copper atoms by gaining electrons. The reaction is as follows:

Cu^2+^ + 2e^−^ → Cu

When more and more Cu^2+^ is reduced to copper atoms at several locations at the bottom electrode, the corresponding cone-shaped copper accumulations of varying height are likely to form randomly within the device, as shown in Figure 5a. These randomly generated copper atom stacks of different heights and sizes will, to a certain extent, change the electric field distribution inside the thin film. According to Liu’s work [26], the strongest electrical field appears at the tip of the highest precipitate. Therefore, driven by the electric field, most Cu ions accumulate at the highest precipitate which results in its fastest growth rate and the first conductive filament connecting the top and bottom electrodes, thereby switching the device from HRS to LRS1, as shown in Figure 5b. As the current further increases, the primary conductive filament (CF) thickens and the other copper atomic accumulations, initially non-bridging, also experience rapid growth, quickly leading to the formation of a second, third, and ultimately more conductive filaments that connect the top and bottom electrodes, which enables the device resistance to be set to LRS2, as shown in Figure 5c. The progression continues until the compliance current is reached, at which point the device transitions to its final LRS3, seen in Figure 5d, and the formation of multiple conductive filaments ultimately leads to the realization of multilevel storage.

To explore the conduction mechanism of the Cu/a-SiC/P^+^-Si structure, the I–V curve in the LRS was redrawn in double logarithmic scale.

**Figure 6:**
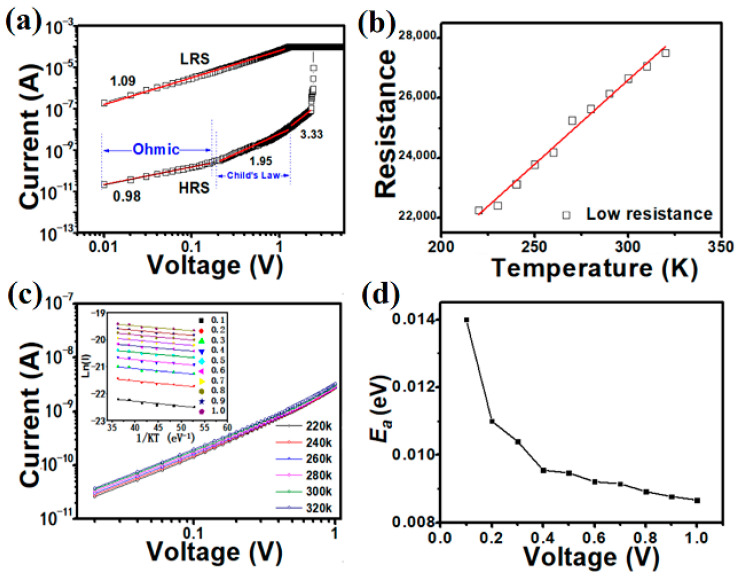
(**a**) I–V characteristics of the Cu/a-SiC/P^+^-Si device in a double natural logarithmic plot. (**b**) The temperature dependence of LRS resistance. (**c**) Temperature dependence of resistance for the HRS from 220 K to 320 K, showing non-metallic conducting behavior. The inset shows an Arrhenius plot of the temperature-dependent current of the HRS. (**d**) The activation energy of the Cu/a-SiC/P^+^-Si device in the HRS, which is decreased with increasing voltage.

Figure 6a shows a straight line with a slope of 1 in the log–log scale for the LRS, which suggests an ohmic characteristic. Figure 6b shows the temperature dependence of resistance in the LRS. The LRS current decreases linearly with increasing temperature, indicating metallic characteristics, as observed in Figure 6b. As is known, the relationship between the resistance of a metal and temperature can be expressed as [9]:

(1) $$R(T)\;=\;R_{0}(1\;+\;\alpha (T-T_{0}))$$

where α is the temperature coefficient. According to Figure 6b, the relationship between the resistances and temperature is linear; according to Equation (1), the temperature coefficient is the slope of the line. The temperature coefficient of the filament is about 2.02 × 10^−3^ K^−1^ in our device. This result is within the copper temperature coefficient range [9,25,27], which proves that the main component of the conducting filament in the Cu/a-SiC/P^+^-Si device is the presence of Cu atoms.

In the HRS, the I–V curve can be fitted as three regions: the ohmic region, Child’s law region, and abruptly increased current region, as seen in Figure 6a. So, the RS behavior can be explained well by the trap-controlled space-charge-limited current (SCLC) model [27]. We also perform temperature-dependent measurements to confirm the proposed SCLC model for the HRS. Figure 6c shows the temperature dependence of the HRS. Note that the current increases with the temperature, which suggests a semiconductor-like conducting behavior [9,23]. Calculated from the slopes of Arrhenius-type plots of the data, the activation Ea in the HRS at a voltage from 0.1 V to 1 V is summarized in the inset of Figure 6c. It is observed that, as seen in Figure 6d, all the $E_{\alpha }$ reduce with increasing voltage, which is a characteristic feature of SCLC [28].

To better evaluate the potential of the Cu/a-SiC/P^+^-Si device for multilevel storage applications, we conducted a series of comparisons on the multilevel storage characteristics of the Cu/a-SiC/P^+^-Si device with those of previous silicon carbide-based resistive random-access memory and oxide-based resistive random-access memory, as presented in Table 1.

According to the statistical data shown in the table, it can be observed that the Cu/a-SiC/P^+^-Si device shares the same resistive switching mechanism as previously reported for SiC-based resistive random-access memory. However, there are some variations in performance due to the different bottom electrode. Although the Cu/a-SiC/P^+^-Si device does not exhibit an ultra-large memory window and ultra-long data retention like Cu/a-SiC/Au or Cu/a-SiC/W, it has still been proven to possess acceptable multilevel storage capability. It realizes four states (2 bits) like the Cu/a-SiC/Au structure, and its more than 500 endurance cycles and over 10^5^ S data retention both demonstrate the stable reliability of the Cu/a-SiC/P^+^-Si device. Compared with the recently reported TiO_x_-based resistive memory, which achieves resistive switching by modulating the bulk concentration of oxygen vacancies, the number of resistance states of the Cu/a-SiC/P^+^-Si device is far fewer than the 32 tunable resistance states of the TiO_x_-based resistive memory. Although the number of achievable resistance states in the Cu/a-SiC/P^+^-Si device is only four, the difference between each state is more than ten times, which makes it easy to distinguish between states in practical applications and reduces the risk of multilevel storage device failure due to state overlap. In contrast, the 32 resistance states of the TiO_x_-based resistive memory device differ by less than two times. Such a large number of continuously adjustable resistance states is more conducive to simulating the weight updates of biological synapses in the human brain. In addition, the Cu/a-SiC/P^+^-Si device exhibits the fastest resistive switching speed (200–300 ns) among the Cu/a-SiC/Au, Cu/a-SiC/W and Au/Al_2_O_3_/TiO_2_/TiO_x_/Au devices. The combination of stability, reliability, and high speed and its relatively ideal multilevel storage capability endow the Cu/a-SiC/P^+^-Si device with significant application potential in future high-speed, high-density memory technologies.

## 4. Summary

In conclusion, we have systematically demonstrated and analyzed the controlled multilevel operation in a Cu/a-SiC/P^+^-Si device. According to the temperature-dependent test results, the formation of Cu conducting filaments is believed to be the reason for the resistance switching from the HRS to the LRS. A controllable filamentary model on the width and the growth by modulating CC is proposed to understand the multilevel behavior. Four different resistance values are achieved in the Cu/a-SiC/P^+^-Si device, and each LRS ratio is sufficient by about 10 times to be discriminated by external circuitry. The good endurance and retention abilities of the four different resistance values are demonstrated, showing the potential application for high-performance multilevel RRAM.

## Figures and Tables

**Table 1:** Comparison with the multilevel storage characteristics of other RRAM devices.

Device	Cu/a-SiC/Au [18]	Cu/a-SiC/W [17]	Cu/a-SiC/P^+^-Si	Ti_2_O_2_ [29]
Switching Mechanism	the formation and rupture of Cu conductive filaments	the formation and rupture of Cu conductive filaments	the formation and rupture of Cu conductive filaments	bulk switching instead of filamentary switching
Solutions	by modulating CC	by modulating CC	by modulating CC	by posing electric field
On/Off	10^7^~10^8^	10^8^~10^9^	10^4^~10^5^	~10
Number of RSs	4	5	4	32
Endurance	Not found	Not found	More than 500 cycles	Not found
Retention	10^9^ s	10^9^ s	10^5^ s	Not found
Switching Speed	Not found	Not found	200~300 ns	500 µs

## Data Availability

The raw data supporting the conclusions of this article will be made available by the authors upon request.
